# Survival Rates for Patients With Barrett High-grade Dysplasia and Esophageal Adenocarcinoma With or Without Human Papillomavirus Infection

**DOI:** 10.1001/jamanetworkopen.2018.1054

**Published:** 2018-08-03

**Authors:** Shanmugarajah Rajendra, Wei Xuan, Neil Merrett, Preeti Sharma, Prateek Sharma, Darren Pavey, Tao Yang, Leonardo D. Santos, Omar Sharaiha, Girish Pande, Peter Cosman, Xiaojuan Wu, Bin Wang

**Affiliations:** 1 Gastro-Intestinal Viral Oncology Group, Ingham Institute for Applied Medical Research, Liverpool, New South Wales, Australia; 2 South Western Sydney Clinical School, University of New South Wales, Kensington, New South Wales, Australia; 3 Department of Gastroenterology & Hepatology, Bankstown-Lidcombe Hospital, South Western Sydney Local Health Network, Bankstown, New South Wales, Australia; 4 Ingham Institute for Applied Medical Research, Liverpool, New South Wales, Australia; 5 Discipline of Surgery, School of Medicine, Western Sydney University, Penrith, New South Wales, Australia; 6 Department of Upper Gastrointestinal Surgery, Bankstown-Lidcombe Hospital, Bankstown, New South Wales, Australia; 7 Division of Gastroenterology and Hepatology, Veterans Affairs Medical Center and University of Kansas School of Medicine, Kansas City, Missouri; 8 SydPath, St Vincent’s Hospital Sydney, Darlinghurst, New South Wales, Australia; 9 Department of Anatomical Pathology, Sydney South West Pathology Service, Liverpool Hospital, Liverpool, New South Wales, Australia; 10 Department of Surgery, Launceston General Hospital, Launceston, Tasmania, Australia; 11 Graduate School of Medicine and Illawara Health and Medical Research Institute, University of Wollongong, Wollongong, New South Wales, Australia; 12 Immunohistochemistry and Molecular Pathology Unit, NSW Health Pathology, Liverpool, New South Wales, Australia

## Abstract

**Importance:**

High-risk human papillomavirus (HPV) has been associated with Barrett dysplasia and esophageal adenocarcinoma. Nevertheless, the prognostic significance of esophageal tumor HPV status is unknown.

**Objective:**

To determine the association between HPV infection and related biomarkers in high-grade dysplasia or esophageal adenocarcinoma and survival.

**Design, Setting, and Participants:**

Retrospective case-control study. The hypothesis was that HPV-associated esophageal tumors would show a favorable prognosis (as in viral-positive head and neck cancers). Pretreatment biopsies were used for HPV DNA determination via polymerase chain reaction, in situ hybridization for E6 and E7 messenger RNA (mRNA), and immunohistochemistry for the proteins p16^INK4A^ and p53. Sequencing of *TP53* was also undertaken. The study took place at secondary and tertiary referral centers, with 151 patients assessed for eligibility and 9 excluded. The study period was from December 1, 2002, to November 28, 2017.

**Main Outcomes and Measures:**

Disease-free survival (DFS) and overall survival (OS).

**Results:**

Among 142 patients with high-grade dysplasia or esophageal adenocarcinoma (126 [88.7%] male; mean [SD] age, 66.0 [12.1] years; 142 [100%] white), 37 were HPV positive and 105 were HPV negative. Patients who were HPV positive mostly had high p16^INK4A^ expression, low p53 expression, and wild-type *TP53*. There were more Tis, T1, and T2 tumors in HPV-positive patients compared with HPV-negative patients (75.7% vs 54.3%; difference, 21.4%; 95% CI, 4.6%-38.2%; *P* = .02). Mean DFS was superior in the HPV-positive group (40.3 vs 24.1 months; difference, 16.2 months; 95% CI, 5.7-26.8; *P* = .003) as was OS (43.7 vs 29.8 months; difference, 13.9 months; 95% CI, 3.6-24.3; *P* = .009). Recurrence or progression was reduced in the HPV-positive cohort (24.3% vs 58.1%; difference, −33.8%; 95% CI, −50.5% to −17.0%; *P* < .001) as was distant metastasis (8.1% vs 27.6%; difference, −19.5%; 95% CI, −31.8% to −7.2%; *P* = .02) and death from esophageal adenocarcinoma (13.5% vs 36.2%; difference, −22.7%; 95% CI, −37.0% to −8.3%; *P* = .01). Positive results for HPV and transcriptionally active virus were both associated with a superior DFS (hazard ratio [HR], 0.33; 95% CI, 0.16-0.67; *P* = .002 and HR, 0.44; 95% CI, 0.22-0.88; *P* = .02, respectively [log-rank test]). Positivity for E6 and E7 mRNA, high p16^INK4A^ expression, and low p53 expression were not associated with improved DFS. On multivariate analysis, superior DFS was demonstrated for HPV (HR, 0.39; 95% CI, 0.18-0.85; *P* = .02), biologically active virus (HR, 0.36; 95% CI, 0.15-0.86; *P* = .02), E6 and E7 mRNA (HR, 0.36; 95% CI, 0.14-0.96; *P* = .04), and high p16 expression (HR, 0.49; 95% CI, 0.27-0.89; *P* = .02).

**Conclusions and Relevance:**

Barrett high-grade dysplasia and esophageal adenocarcinoma in patients who are positive for HPV are distinct biological entities with a favorable prognosis compared with viral-negative esophageal tumors. Confirmation of these findings in larger cohorts with more advanced disease could present an opportunity for treatment de-escalation in the hope of reducing toxic effects without deleteriously affecting survival.

## Key Points

**Question:**

What is the prognostic significance of esophageal tumor human papillomavirus (HPV) status?

**Findings:**

In this case-control study involving 142 patients with Barrett high-grade dysplasia and esophageal adenocarcinoma, HPV positivity was associated with a significantly improved disease-free survival compared with viral negativity. Recurrence and progression were reduced in the HPV-positive cohort as were distant metastasis and death from esophageal adenocarcinoma.

**Meaning:**

Barrett high-grade dysplasia and esophageal adenocarcinoma in patients who are HPV positive have a favorable prognosis compared with viral-negative esophageal tumors and may benefit from treatment de-escalation.

## Teaser

This case-control study compares disease-free and overall survival among patients with Barrett high-grade dysplasia or esophageal adenocarcinoma with vs without underlying human papillomavirus (HPV) infection demonstrated on biopsy.

## Introduction

Esophageal adenocarcinoma (EAC) is one of the fastest-growing and deadliest cancers in the Western world,^1,2^ although recently the rate of increase has diminished and possibly plateaued in the United States and Sweden.^3,4^ Currently, Barrett esophagus (BE) is the only recognized visible precursor lesion for EAC. Intriguingly, these high rates of EAC have occurred against a backdrop of progressive reduction in the risk estimate of malignancy associated with BE.^5^ In this regard, our discovery of a strong association of transcriptionally active high-risk human papillomavirus (HPV) with a subset of Barrett dysplasia (BD) and EAC^5^ may be relevant.

Increasing high-risk HPV viral load and integration status has been linked with more severe disease along the Barrett metaplasia-dysplasia-adenocarcinoma sequence.^6^ Moreover, treatment failure after endoscopic ablation of BD or EAC is predicted by persistent high-risk HPV infection and overexpression of the p53 gene (*TP53*).^7^ We also discovered that HPV-positive EAC is molecularly distinct from HPV-negative EAC, indicating different biological mechanisms of tumor genesis.^8^ Hybrid sequences containing HPV-16 and the human genome have been identified, indicating a host-viral interaction.^8^ Aberrations of the retinoblastoma protein (pRb) pathway, ie, upregulation of p16^INK4A^ and downregulation of pRb, as well as wild-type *TP53* are hallmarks of active HPV involvement in BD and EAC. Additionally, the transformation-specific combination of high p16 expression and low pRb expression, which is a feature of HPV-driven lesions, has been identified in a significant proportion of virally positive BD and EAC.^9^

Ability to detect HPV in earlier negative studies and more recent investigations may have been hampered by poor tissue classification and suboptimal testing methods. This is further exacerbated by the low HPV viral load in esophageal tissue. Some of these misclassified negative studies only used nondysplastic BE. Metaplastic tissue is not associated with HPV. Racial or geographic variations can also account for this anomaly.^10,11,12,13,14^ Interestingly, a systematic review has reported HPV prevalence rates of 35% in 174 patients with EAC, similar to our findings.^15^ Another systematic review that included 19 studies concluded that the pooled prevalence of HPV in EAC was 13%, suggesting the low prevalence rate may have been caused by small sample sizes and compromised detection methods.^16^

It is well documented that patients with HPV-positive head and neck squamous cell carcinoma (HNSCC) have an improved rate of overall survival (OS) (hazard ratio [HR], 0.7; 95% CI, 0.5-1.0) and a reduced risk of recurrence (HR, 0.5; 95% CI, 0.4-0.7) compared with viral-negative tumors.^17^ A meta-analysis reported a 74% improved disease-free survival (DFS) and 53% better OS in HPV-associated HNSCC vs HPV-negative HNSCC.^18^ We therefore hypothesized that HPV-associated EAC and high-grade dysplasia (HGD) would show a similar favorable prognosis compared with HPV-negative esophageal lesions.

## Methods

### 

### Study Design and Population

This retrospective case-control study followed the Strengthening the Reporting of Observational Studies in Epidemiology (STROBE) reporting guideline. Eligible patients were those with HGD or EAC (Siewert classification type I or II) deemed suitable for treatment, ie, for endotherapy (endoscopic mucosal resection [EMR] and/or radiofrequency ablation [RFA]) or esophagectomy with or without neoadjuvant chemotherapy and/or radiotherapy. The study period was from December 1, 2002, to November 28, 2017, and patients were enrolled from a tertiary referral center, Bankstown-Lidcombe Hospital, Sydney, Australia (n = 139), and a regional health care center, Launceston General Hospital, Launceston, Tasmania, Australia (n = 3). Demographic and clinical data were obtained from a prospectively maintained database. Inclusion and exclusion criteria have been previously documented.^9^ Pretreatment tissue was prospectively collected in 94 patients (both fresh frozen and formalin-fixed paraffin-embedded [FFPE]) and retrospectively retrieved (FFPE only) from the remaining 48 patients. Oral and written informed consent were obtained from participants prior to the investigation. This study was approved by the Human Research Ethics Committee, Tasmania, and South Western Sydney Local Health Network.

### Endotherapy and/or Esophagectomy

Staging endoscopic ultrasound examination was performed on all patients. Patients with nodular or ulcerating lesions without lymph node involvement underwent EMR. Apart from endoscopic ultrasound, positron emission tomography, computed tomography, and laparoscopy were performed as staging investigations in patients considered candidates for esophagectomy. Circumferential and focal RFA were used to ablate flat lesions to achieve complete eradication. Patients were scheduled to return for 3-, 6-, 9-, and 12-month follow-up endoscopy and biopsy. Residual lesions were ablated and nodules subjected to EMR.

Patients with adenocarcinoma invading beyond the muscularis mucosa into the submucosa (T1b lesions) were excluded from endotherapy and underwent esophagectomy with D2 lymph node dissection. Siewert type I tumors were subjected to Ivor Lewis esophagogastrectomy and Siewert type II cancers to either radical total gastrectomy including lower esophagus or Ivor Lewis esophagectomy depending on the patient’s build.

Staging was performed according to the seventh edition of the *Cancer Staging Manual* by the American Joint Committee on Cancer.^19^ Patients with locally advanced disease (T3 or T4 N0 or any T stage with N1) were potential candidates for neoadjuvant or adjuvant treatment. After treatment, repeat staging was done to exclude metastatic disease prior to proceeding to esophagectomy. As such, 14 patients did not undergo esophagectomy due to tumor-related factors or patient refusal. Nevertheless, they were included in the analysis as they had undergone radiotherapy and/or chemotherapy.

### Laboratory Studies

Detection of HPV in genomic DNA extracted from fresh frozen or formalin-fixed biopsy tissue was performed by nested polymerase chain reaction (PCR) amplification of a conserved viral L1 gene using MY09 and MY11 and GP5^+^ and GP6^+^ primers for both high-risk and low-risk HPV as previously published.^5^ To minimize contamination, separate rooms were used for reaction preparation, template handling, performing nested reactions, and post-PCR analysis. Routine decontamination by UV irradiation was performed in the DNA-free PCR hood before each run. To guard against systematic contamination of PCR reagent, appropriate positive (HPV-16–positive cervical cancer) and negative (deionized water and PCR master mix without template) controls were included in each step of the PCR process. Genotypes of HPV were determined by sequencing.^5^ Real-time PCR assays measuring HPV E6 and E7 copy numbers were performed to determine viral load using genotype-specific HPV-16 and HPV-18 primers.^6^

In situ hybridization (ISH) of RNA for high-risk HPV-16 and HPV-18 E6 and E7 messenger RNA (mRNA) was performed manually using the RNAscope 2.5 High-Definition Assay (Advanced Cell Diagnostics Inc) with a cocktail of probes targeting 18 high-risk HPV types (HPV types 16, 18, 26, 31, 33, 35, 39, 45, 51, 52, 53, 56, 58, 59, 66, 68, 73, and 82) according to the manufacturer’s instructions.^9^ Negative and positive controls were probes recognizing the bacterial gene dapB and the endogenous ubiquitin C mRNA, respectively. In addition, cervical cancer tissue, HNSCC samples, BE and BD, EAC, and esophageal squamous cell carcinoma (ESCC), which all had detectable transcriptionally active HPV (DNA positive by PCR and the presence of ≥1 of 2 markers of biological activity, ie, E6 and E7 mRNA and/or p16^INK4A^), served as positive controls. We used HNSCC, BE and BD, EAC, and ESCC devoid of virus as negative controls. Positivity was defined as the presence of punctuate cytoplasmic and/or nuclear staining that exceeded the dapB (negative control) signal. Expression of the p16^INK4A^ and p53 proteins was assessed by immunohistochemistry (IHC) on FFPE tissue using EnVision FLEX Mini Kits and CINtec Histology Kits (monoclonal mouse anti–human p16^INK4A^ antibody, clone E6H4) (mtm laboratories), respectively, using appropriate positive and negative controls.^9^ All IHC scoring of slides was independently performed by 2 experienced gastrointestinal pathologists blinded to the virological status and the clinical outcome of patients. For p16^INK4A^, at least moderate staining of both nucleus and cytoplasm in more than 25% of esophageal dysplastic or tumor tissue was considered as p16 overexpression. Staining of 25% or less was deemed low expression. Using this criterion, we have demonstrated that high p16 expression is associated with HPV-associated BD and EAC with reasonable sensitivity and specificity as others have in HNSCC.^9,20^ In the case of p53, intense nuclear staining of more than 10% of esophageal cells was considered overexpression.^7,9^ Mutations of *TP53* were confirmed by sequencing of the gene using the semiconductor-based Ion Torrent sequencing platform (ThermoFisher) according to the manufacturer’s instructions as previously published.^9^

### Study End Points and Statistical Analysis

The primary end points were DFS from the time of diagnosis to the date of first local, regional, or distant failure and OS, defined as time between diagnosis to the date of death or last follow-up.

Differences between HPV-positive and HPV-negative cases in regard to baseline characteristics were assessed using the 2-sample *t* test for comparing the mean values between the 2 groups in regard to all numerical data. A χ^2^ analysis was used for evaluating the association between the binary measurements in the viral-positive and viral-negative groups. Survival analysis was conducted using the Kaplan-Meier method to estimate the DFS and OS of the 5 HPV variables, ie, viral DNA status, transcriptionally active HPV, E6 and E7 mRNA, p16, and p53. The log-rank test was used to analyze the association between the HPV variables with DFS and OS. Cox proportional hazards regression models were used to estimate the importance of these biomarkers for DFS and OS after adjusting for age, sex, body mass index (calculated as weight in kilograms divided by height in meters squared), history of smoking, excess alcohol use, medications (proton-pump inhibitors, nonsteroidal anti-inflammatory drugs, or statins), T stage, N stage, surgical or endoscopic mucosal resection margin status, degree of tumor differentiation, and neoadjuvant or adjuvant treatment (model 2). Because viral status itself was associated with disease severity, adjusting for stage and treatment (chemotherapy and/or radiotherapy) may have prevented identification of prognostic effects derived exclusively from the 5 HPV variables. Therefore, additional Cox regression models were applied with all of the above covariates but excluding T and N stages as well as chemotherapy and radiotherapy (model 3). All statistical tests were performed using SAS statistical software version 9.4 (SAS Institute Inc), and the level of significance was set at .05 (2-sided).

## Results

### Patient Characteristics

A total of 142 patients were tested for HPV status, p16^INK4A^ IHC, and E6 and E7 mRNA ISH (eFigure 1 in the Supplement). Of 142 patients (126 [88.7%] male; mean [SD] age, 66.0 [12.1] years; 142 [100%] white), 37 were HPV positive and 105 were HPV negative. Mean (SD) follow-up time was 33.4 (28.0) months (range, 2-159 months) for the whole study population and 43.8 (29.4) months (range, 3-159 months) for survivors.

### HPV DNA, E6 and E7 mRNA, p16^INK4A^, and p53 Status

Polymerase chain reaction analysis of HPV DNA was performed on all 142 patients with esophageal lesions (38 HGD and 104 EAC). Thirty-seven patients (11 with HGD and 26 with EAC) had positive results for HPV DNA; 33 had HPV-16, 1 had HPV-18, and the remaining 3 had low-risk types 6 and 11. No multiple genotypes were detected in the same patient. Amplifiable β-globin gene was present in all specimens, and median (range) viral load was 0.1 copy per 10 cell genomic DNA (0-1.12 copies per 10 cell genome). All specimens with measurable viral load revealed coherence between genotypes found on MY09 and MY11 and GP5^+^ and GP6^+^ PCR and those found in E6 and E7 analysis. In the 142 HGDs and EACs assessed, 33 of 34 high-risk HPV (types 16 and 18) (97.1%) had detectable viral load. Among the 37 DNA-positive lesions, 18 (48.7%) had E6 and E7 mRNA detection by ISH and 19 (51.4%) overexpressed p16^INK4A^. Eleven samples (29.7%) were positive for all 3 markers.

Demographic, clinical, and pathological data are compared between HPV-positive and HPV-negative individuals with esophageal lesions in Table 1. Overexpression of p16^INK4A^ and E6 and E7 mRNA presence were significantly greater in the HPV-positive group compared with the viral-negative cohort. Of note, HPV-positive patients had more early-stage (Tis, T1, and T2) esophageal lesions compared with viral-negative patients (75.7% vs 54.3%; difference, 21.4%; 95% CI, 4.6%-38.2%; *P* = .02), but nodal stage was similar between the groups. Most were stage N0 or N1 in both the HPV-positive (89.2%) and viral-negative (89.5%) patients with HGD and EAC. No significant differences were detected in any of the other clinical or pathological baseline characteristics. In the transcriptionally active HPV-positive group (HPV positive with p16^INK4A^, HPV positive with E6 and E7 mRNA, or HPV positive with both p16^INK4A^ and E6 and E7 mRNA), the patients were significantly younger than the biologically inactive virus group (HPV positive without p16^INK4A^ and/or E6 and E7 mRNA) (61.4 vs 67.2 years; difference, −5.8 years; 95% CI, −10.6 to −1.0 years; *P* = .02) (eTable in the Supplement).

**Table 1.** Demographic and Clinical Characteristics of the Study Population and Associated Tumors According to Patient Group

Characteristic	No. (%)^a^		*P* Value^b^
	Patients With HPV-Positive HGD or EAC (n = 37)	Patients With HPV-Negative HGD or EAC (n = 105)	
Sex			
Male	33 (89.2)	93 (88.6)	.92
Female	4 (10.8)	12 (11.4)	
Age, mean (SD) (range), y	65.2 (12.4) (33.0-89.0)	66.2 (12.0) (32.0-90.0)	.65
Body mass index, mean (SD)^c^	27.7 (5.7)	27.0 (5.1)	.53
Ever smoked	24 (64.9)	77 (73.3)	.33
Smoked >10 pack-years^d^	22/24 (91.7)	69/76 (90.8)	.90
Smoked >20 pack-years^d^	15/24 (62.5)	48/76 (63.2)	.95
Alcohol intake	26/36 (72.2)	75 (71.4)	.93
Excess alcohol^e^	8/35 (22.9)	18 (17.1)	.45
Proton-pump inhibitors	28 (75.7)	75 (71.4)	.62
Nonsteroidal anti-inflammatory drugs	11 (29.7)	23 (21.9)	.34
Statins	11 (29.7)	31 (29.5)	.98
Esophagitis	4/36 (11.1)	14/104 (13.5)	.72
Hiatal hernia	21/36 (58.3)	49/101 (48.5)	.31
Resection margin			
R0	26/29 (89.7)	61/75 (81.3)	.30
R1 or R2	3/29 (10.3)	14/75 (18.7)	
Histology, degree of differentiation			
Well	3 (8.1)	5 (4.8)	.22
Moderate	14 (37.8)	31 (29.5)	
Poor	5 (13.5)	32 (30.5)	
Not documented	15 (40.5)	37 (35.2)	
Treatment endotherapy, EMR and RFA	17 (46.0)	41 (39.1)	.46
Esophagectomy	21 (56.8)	54 (51.4)	.58
Pathologic T stage^f^			
Tis, T1, or T2	28 (75.7)	57 (54.3)	.02
T3 or T4	9 (24.3)	48 (45.7)	
Pathologic N stage^f^			
N0-N1	33 (89.2)	94 (89.5)	.95
N2-N3	4 (10.8)	11 (10.5)	
Pathologic M stage^f^			
M0	36 (97.3)	99 (94.3)	.47
M1	1 (2.7)	6 (5.7)	
Radiotherapy	8 (21.6)	28 (26.7)	.54
Chemotherapy	13 (35.1)	39 (37.1)	.83
p16^INK4a^ overexpression	19 (51.4)	34 (32.4)	.04
E6 and E7 mRNA positivity	18 (48.7)	6 (5.7)	<.001
Low p53 expression	23 (62.2)	40 (38.1)	.01

Abbreviations: EAC, esophageal adenocarcinoma; EMR, endoscopic mucosal resection; HGD, high-grade dysplasia; HPV, human papillomavirus; mRNA, messenger RNA; RFA, radiofrequency ablation.

^a^ Denominators are listed when sample does not equal full sample size.

^b^ Differences between HPV-positive vs HPV-negative cases in regard to baseline characteristics were assessed using 2-sample *t* test for all numerical data and χ^2^ analysis for binary measurements.

^c^ Calculated as weight in kilograms divided by height in meters squared.

^d^ A pack-year indicates smoking 1 pack of cigarettes per day for a year.

^e^ Excess alcohol intake was defined as more than 21 units/wk for men and more than 14 units/wk for women.

^f^ TNM classification as per the American Joint Committee on Cancer’s *Cancer Staging Manual,* 7th Edition.

### HPV Status and Survival

Table 2 depicts the mean follow-up, DFS, OS, and recurrence or progression rate for the HPV-positive patients with HGD and EAC and the viral-negative groups. Mean (SD) DFS was 40.3 (33.8) months in the HPV-positive esophageal lesion group and 24.1 (25.5) months in the HPV-negative cohort (difference, 16.2 months; 95% CI, 5.7-26.8 months; *P* = .003). Similarly, mean (SD) OS was 43.7 (32.9) months in the viral-positive group compared with 29.8 (25.3) months in the HPV-negative patients (difference, 13.9 months; 95% CI, 3.6 to 24.3 months; *P* = .009). As expected, recurrence or progression was much reduced in the HPV-positive cohort compared with the HPV-negative cohort (9 of 37 [24.3%] vs 61 of 105 [58.1%]; difference, −33.8%; 95% CI, −50.5% to −17.0%; *P* < .001). Recurrence per se was almost a third less in the HPV-positive patients in comparison with HPV-negative patients (6 of 37 [16.2%] vs 46 of 105 [43.8%]; difference, −27.6%; 95% CI, −42.8% to −12.4%; *P* = .003). There appeared to be better (although nonsignificant) local and regional control for the HPV-positive patients compared with viral-negative individuals (6 of 37 [16.2%] vs 32 of 105 [30.5%]; difference, −14.3%; 95% CI, −29.0% to 0.5%; *P* = .09). Significantly, reduced distant metastasis was observed in the HPV-positive group (3 of 37 [8.1%] vs 29 of 105 [27.6%]; difference, −19.5%; 95% CI, −31.8% to −7.2%; *P* = .02) as were deaths from EAC (5 of 37 [13.5%] vs 38 of 105 [36.2%]; difference, −22.7%; 95% CI, −37.0% to −8.3%; *P* = .01).

**Table 2.** Comparison of Survival, Disease Relapse and Progression, and Site of Failure in HPV-Positive and HPV-Negative Patients

Characteristic	No. (%)		*P* Value^a^
	Patients With HPV-Positive HGD or EAC (n = 37)	Patients With HPV-Negative HGD or EAC (n = 105)	
Disease-free survival, mean (SD), mo	40.3 (33.8)	24.1 (25.5)	.003
Overall survival, mean, mo	43.7 (32.9)	29.8 (25.3)	.009
Survival status (alive at last follow-up)	26 (70.3)	58 (55.2)	.11
Recurrence or progression	9 (24.3)	61 (58.1)	<.001
Recurrence	6 (16.2)	46 (43.8)	.003
Local-regional failure	6 (16.2)	32 (30.5)	.09
Distant metastases	3 (8.1)	29 (27.6)	.02
Death due to EAC	5 (13.5)	38 (36.2)	.01

Abbreviations: EAC, esophageal adenocarcinoma; HGD, high-grade dysplasia; HPV, human papillomavirus.

^a^ Differences between HPV-positive vs HPV-negative cases in regard to characteristics were assessed using 2-sample *t* test for all numerical data and χ^2^ analysis for binary measurements.

Kaplan-Meier graphs for DFS and OS of patients categorized by HPV, transcriptionally active virus, E6 and E7 mRNA, and high expression of p16 and low expression of p53 are shown in the Figure and in eFigure 1, eFigure 2, and eFigure 3 in the Supplement. The log-rank test revealed that HPV status and transcriptionally active viral presence were individually associated with a superior DFS of 67% (HR, 0.33; 95% CI, 0.16-0.67; *P* = .002) and 56% (HR, 0.44; 95% CI, 0.22-0.88; *P* = .02), respectively (model 1 in Table 3). Conversely, positive status for E6 and E7 mRNA, high expression of p16^INK4A^, and low expression of p53 were not associated with improved DFS. Subsequently, Cox proportional hazards models were used to estimate the importance of these biomarkers for DFS and OS after adjusting for all the 14 variables mentioned under Study End Points and Statistical Analysis. This revealed statistically superior DFS for HPV (HR, 0.39; 95% CI, 0.18-0.85; *P* = .02), biologically active virus (HR, 0.36; 95% CI, 0.15-0.86; *P* = .02), E6 and E7 mRNA (HR, 0.36; 95% CI, 0.14-0.96; *P* = .04), and high p16 expression (HR, 0.49; 95% CI, 0.27-0.89; *P* = .02) (model 2 in Table 3).

**Figure.**
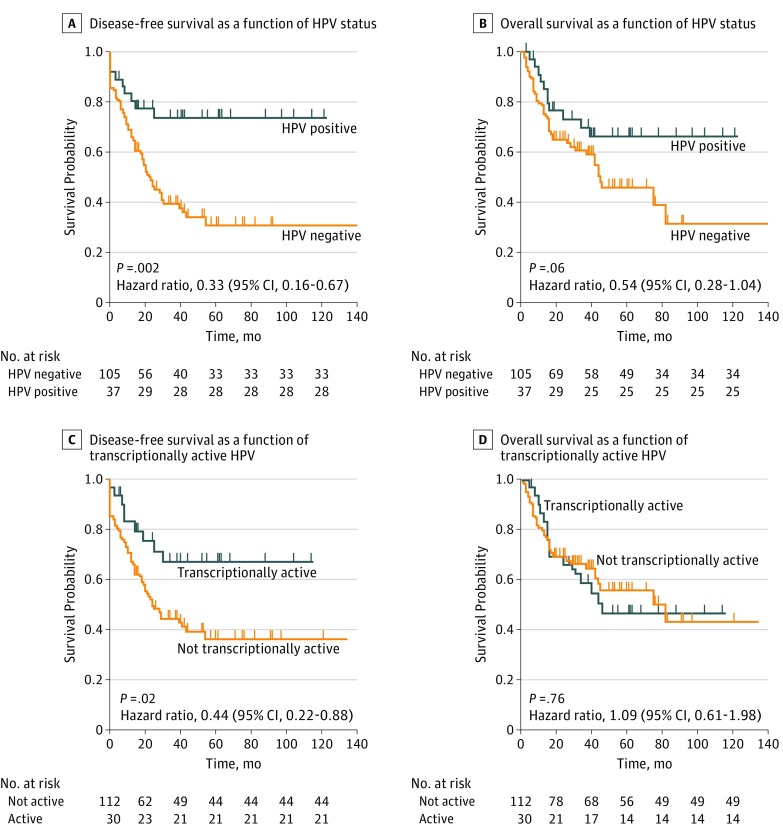
Survival Among Patients With High-Grade Dysplasia or Esophageal Adenocarcinoma as a Function of Human Papillomavirus (HPV) and Transcriptionally Active HPV Of 142 patients with either high-grade dysplasia or esophageal adenocarcinoma, 37 were HPV positive and 105 were HPV negative (A and B). Thirty patients had transcriptionally active HPV and 112 patients were HPV negative or had HPV that was not transcriptionally active (C and D).

**Table 3.** Log-Rank and Multivariate Disease-Free Survival Analysis (Cox Regression)

Characteristic	Model 1^a^		Model 2^b^		Model 3^c^	
	Disease-Free Survival, HR (95% CI)	Unadjusted *P* Value	Disease-Free Survival, HR (95% CI)	Adjusted *P* Value	Disease-Free Survival, HR (95% CI)	Adjusted *P* Value
HPV positive	0.33 (0.16-0.67)	.002	0.39 (0.18-0.85)	.02	0.36 (0.17-0.77)	.009
Transcriptionally active HPV positive	0.44 (0.22-0.88)	.02	0.36 (0.15-0.86)	.02	0.31 (0.13-0.72)	.006
E6 and E7 mRNA positive	0.50 (0.24-1.05)	.07	0.36 (0.14-0.96)	.04	0.32 (0.12-0.83)	.02
High p16 expression	0.76 (0.46-1.25)	.28	0.49 (0.27-0.89)	.02	0.59 (0.34-1.02)	.06
Low p53 expression	0.85 (0.53-1.37)	.51	0.89 (0.53-1.51)	.66	0.84 (0.51-1.39)	.50

Abbreviations: HPV, human papillomavirus, HR, hazard ratio; mRNA, messenger RNA.

^a^ Model 1 was a univariate analysis on each characteristic with disease-free survival.

^b^ In model 2, each characteristic was analyzed in a multivariate Cox regression separately, adjusted by the following covariates: age, sex, body mass index (calculated as weight in kilograms divided by height in meters squared), ever smoked, excess alcohol, proton-pump inhibitor use, nonsteroidal anti-inflammatory ingestion, statin use, T stage, N stage, R0 resection margin, chemotherapy and radiotherapy, and tumor differentiation.

^c^ Model 3 was the same as model 2 but excluded the following covariates in the adjustment: T stage, N stage, chemotherapy, and radiotherapy.

In regard to OS, HPV status was not statistically associated with improved prognosis (HR, 0.54; 95% CI, 0.28-1.04; log-rank *P* = .06) (model 1 in Table 4).

**Table 4.** Log-Rank and Multivariate Overall Survival Analysis (Cox Regression)

Characteristic	Model 1^a^		Model 2^b^		Model 3^c^	
	Overall Survival, HR (95% CI)	Unadjusted *P* Value	Overall Survival, HR (95% CI)	Adjusted *P* Value	Overall Survival, HR (95% CI)	Adjusted *P* Value
HPV positive	0.54 (0.28-1.04)	.06	1.06 (0.46-2.46)	.89	0.64 (0.31-1.31)	.22
Transcriptionally active HPV positive	1.09 (0.61-1.98)	.76	1.80 (0.80-4.05)	.16	1.01 (0.52-1.98)	.98
E6 and E7 mRNA positive	1.23 (0.66-2.28)	.52	1.09 (0.47-2.51)	.84	1.02 (0.49-2.14)	.96
High p16 expression	1.32 (0.79-2.22)	.29	1.26 (0.66-2.40)	.48	1.06 (0.60-1.89)	.84
Low p53 expression	1.27 (0.76-2.12)	.37	1.62 (0.90-2.93)	.11	1.32 (0.76-2.32)	.33

Abbreviations: HPV, human papillomavirus; HR, hazard ratio; mRNA, messenger RNA.

^a^ Model 1 was a univariate analysis on each characteristic with overall survival.

^b^ In model 2, each characteristic was analyzed in a multivariate Cox regression separately, adjusted by the following covariates: age, sex, body mass index (calculated as weight in kilograms divided by height in meters squared), ever smoked, excess alcohol, proton-pump inhibitor use, nonsteroidal anti-inflammatory ingestion, statin use, T stage, N stage, R0 resection margin, chemotherapy and radiotherapy, and tumor differentiation.

^c^ Model 3 was the same as model 2 but excluded the following covariates in the adjustment: T stage, N stage, chemotherapy, and radiotherapy.

As viral status itself was associated with disease severity and the likelihood of too many covariates diluting the multivariate models, further analysis of these HPV-related factors was undertaken using Cox models without adjustment for stage or treatment options (model 3 in Table 3 and Table 4). This resulted in a significantly enhanced DFS for HPV DNA positivity (HR, 0.36; 95% CI, 0.17-0.77; *P* = .009), presence of transcriptionally active virus (HR, 0.31; 95% CI, 0.13-0.72; *P* = .006), and E6 and E7 mRNA detection (HR, 0.32; 95% CI, 0.12-0.83; *P* = .02) (model 3 in Table 3). Although the HR was lower for all 5 of these variables in relation to OS, none were statistically significant (model 3 in Table 4).

### Next-Generation Sequencing of *TP53*

Of 142 HGD and EAC specimens, 132 (93.0%) were successfully sequenced, and in 104 of 132 (78.8%) p53 IHC and sequencing data matched. Seventy-three (55.3%) harbored mutated *TP53* and 59 (44.7%) had wild type. In the 73 specimens with *TP53* mutations, 50 (68.5%) were missense mutations present in the DNA binding domain (exons 5-8; aa102-292), 2 (2.7%) were missense mutations in the oligomerization domain (exons 9-10), and 2 contained frameshift mutations in exons 5 and 8 that induced a loss of both DNA binding and oligomerization domains. In 35 of 37 HPV-positive patients with HGD and EAC with successful sequencing, *TP53* mutations were detected in only 9 patients (25.7%) compared with 64 of 97 (66.0%) in the viral-negative group (difference, −40.3%; 95% CI, −57.5% to −23.0%; *P* < .001 [Fisher exact test]).

## Discussion

This is the first study, to our knowledge, to show improved survival associated with HPV-positive HGD and EAC. Esophageal lesional HPV status and associated viral transcriptional markers, ie, E6 and E7 mRNA (gold standard) and p16^INK4A^ (surrogate marker) are associated with improved DFS in patients with HGD and EAC. It was mainly due to a reduction in distant metastasis and possibly better local and regional control in HPV-positive patients compared with HPV-negative patients. This resulted in a lower mortality from EAC. Mean duration of OS was again significantly improved in the HPV-positive group compared with the HPV-negative group. Nevertheless, the association of HPV status with OS failed to reach significance by the log-rank test. Although 26 of 37 HPV-positive individuals (70.3%) were alive at the end of the follow-up period compared with 58 of 105 HPV-negative individuals (55.2%), this was not statistically significant, possibly because of the modest sample size and associated comorbidities. These findings are somewhat similar to the data in head and neck cancers. Human papillomavirus–positive HNSCCs have improved survival and lower rate of local and regional recurrence compared with HPV-negative head and neck cancers. No significant differences were detected in distant metastases, possibly because the studies were insufficiently powered.^21,22,23^ In ESCC, it has been reported that patients with p16-positive cancers had superior 5-year OS and PFS rates compared with patients with p16-negative cancers.^24^ Similarly, Kumar and colleagues^25^ found that ESCC patients with p16-positive tumors subjected to neoadjuvant chemotherapy had better complete remission rates than the p16-negative group. Conversely, in a recent publication, HPV, p16, and p53 were not found to be prognostic factors in ESCC.^26^

The results of this study are consistent with our previous work that demonstrated HPV-positive and HPV-negative EAC are distinct diseases just as in HNSCC. A previous study found that HPV-positive EAC harbored approximately 50% fewer nonsilent somatic mutations than HPV-negative EAC.^8^ Moreover, *TP53* aberrations are less frequent in HPV-positive BD and EAC compared with viral-negative esophageal lesions.^7,8,9^ Prior studies from our group have also demonstrated that HPV-positive BD and EAC are mostly wild-type *TP53*.^7,9^ In this study, we similarly found that only a quarter of HPV-positive HGD and EAC lesions harbored *TP53* mutations vs two-thirds of HPV-negative HGD and EAC having the same molecular aberration. Although patients with EAC who have p53 mutations have been shown to have reduced OS in a recent meta-analysis, we could not confirm the same in our study.^27^ Possible reasons include the small sample size and other nonviral interactions involving p53 function, including smoking (which in this study was high in both HPV-positive and HPV-negative patients) and alcohol use.^28^ Not surprisingly, comorbidity, especially smoking related, also influences survival negatively.^29^ Response to chemotherapy in the presence of other antiapoptotic proteins not analyzed in this study (eg, Bcl-2 and Bcl-xL, which are associated with both chemotherapy and radiation resistance) could be another reason for this discrepancy.^30,31,32^

Patients whose HGD and EAC harbored transcriptionally active HPV (n = 30) were significantly younger (mean [SD] age, 61.4 [11.9] years) compared with patients with esophageal lesions devoid of biologically active virus (n = 112) (mean [SD] age, 67.2 [11.9] years). This is in keeping with findings from our previous small study on exome sequencing of HPV-positive and HPV-negative patients.^8^ Interestingly, three-quarters of HPV-positive esophageal lesions were early T stage (Tis, T1, and T2) compared with slightly more than half of the HPV-negative group. Nevertheless, the vast majority of both cohorts consisted of nodal stage N0 or N1 (89.2% of HPV-positive patients and 89.5% of viral negative patients), which is probably indicative of patient selection for endotherapy or esophagectomy with curative intent. Our results concur somewhat with data from HNSCC, another malignant neoplasm in which a subset are HPV driven. Patients with HPV-positive HNSCC are generally 10 years younger^33^ and have more early T-stage but advanced N-stage disease, although they respond better to treatment and have a better outcome than patients with HPV-negative HNSCC.^34,35^

The determination of HPV presence in BD or EAC can be difficult using FFPE specimens. While PCR is more sensitive than ISH for HPV DNA detection, it risks false-positivity. Thus, in addition to PCR for viral DNA, we also included E6 and E7 mRNA transcript analysis by ISH, which is a reliable marker of HPV involvement, and IHC for p16 overexpression, a surrogate marker for HPV infection. Sequencing and IHC for p53 were also undertaken given their importance in BD and EAC progression.^36^ Central reporting of biopsy specimens by experienced gastrointestinal pathologists was an added strength.

### Limitations

This investigation was retrospective in nature and the study sample was small. The case-control nature of the study introduces biases pertaining to selection, information, and observation as well as confounding. As HPV status was not known at the time of enrollment and treatment decision, it minimized both selection and observer bias. Measurement bias was addressed by blinding the scientist and pathologists to the clinical and virological status of the patients and to treatment outcome. Confounding was mitigated with adjustment for potential confounders in the multivariate statistical analysis. The independence of prognostic effects of the 5 dichotomous variables, HPV DNA positivity, transcriptionally active HPV, E6 and E7 mRNA detection, and p16 and p53 overexpression, is important. Nevertheless, we could not include these variables in a single multivariate model as they were intercorrelated. Thus, we were unable to identify the independent effects of these factors. Some of the specimens analyzed were more than 10 years old, which increases the risk of DNA and RNA invalidity. Moreover, IHC analysis is subjective and lacks uniform scoring systems, which can hamper reproducibility between studies.

## Conclusions

If these findings of a favorable prognosis of HPV-positive HGD and EAC are confirmed in larger cohorts with more advanced disease, it presents an opportunity for treatment de-escalation in the hope of reducing toxic effects without deleteriously affecting survival.

## Notes

Supplement. **eFigure 1.** Flow Diagram Showing HPV DNA and Transcriptional Marker Status of Enrolled Study Patients **eFigure 2.** E6 and E7 mRNA Status and p16 Overexpression **eFigure 3.** p53 Expression **eTable.** Demographic and Clinical Characteristics of the Study Population and Associated Tumors According to Patient Group
