# Supplementary material for: Survival Rates for Patients With Barrett High-grade Dysplasia and Esophageal Adenocarcinoma With or Without Human Papillomavirus Infection
Source: JAMA Netw Open. 2018 Aug 3;1(4):e181054. doi: 10.1001/jamanetworkopen.2018.1054 (PMC6324261; doi:10.1001/jamanetworkopen.2018.1054)
Supplement: Supplement. — eFigure 1. Flow Diagram Showing HPV DNA and Transcriptional Marker Status of Enrolled Study Patients eFigure 2. E6 and E7 mRNA Status and p16 Overexpression eFigure 3. p53 Expression eTable. Demographic and Clinical Characteristics of the Study Population and Associated Tumors According to Patient Group [file jamanetwopen-1-e181054-s001.pdf]

Supplementary Online Content

Rajendra S, Xuan W, Merrett N, et al. Survival Rates for Patients With Barrett High-grade Dysplasia and Esophageal Adenocarcinoma With or Without Human Papillomavirus Infection. *JAMA Netw Open.* 2018;1(4):e181054. doi:10.1001/jamanetworkopen.2018.1054

Supplement. eFigure 1. Flow Diagram Showing HPV DNA and Transcriptional Marker Status of Enrolled Study Patients

eFigure 2. E6/E7 mRNA Status and p16 Overexpression

eFigure 3. p53 Expression

eTable. Demographic and Clinical Characteristics of the Study Population and Associated Tumors According to Patient Group

This supplementary material has been provided by the authors to give readers additional information about their work.

© 2018 Rajendra S et al. *JAMA Network Open.*

eFigure 1. Flow diagram showing HPV DNA and transcriptional marker status of enrolled study patients

Flow diagram showing HPV DNA and transcriptional marker status of enrolled study patients. HPV, human papillomavirus, ISH, in-situ hybridization.

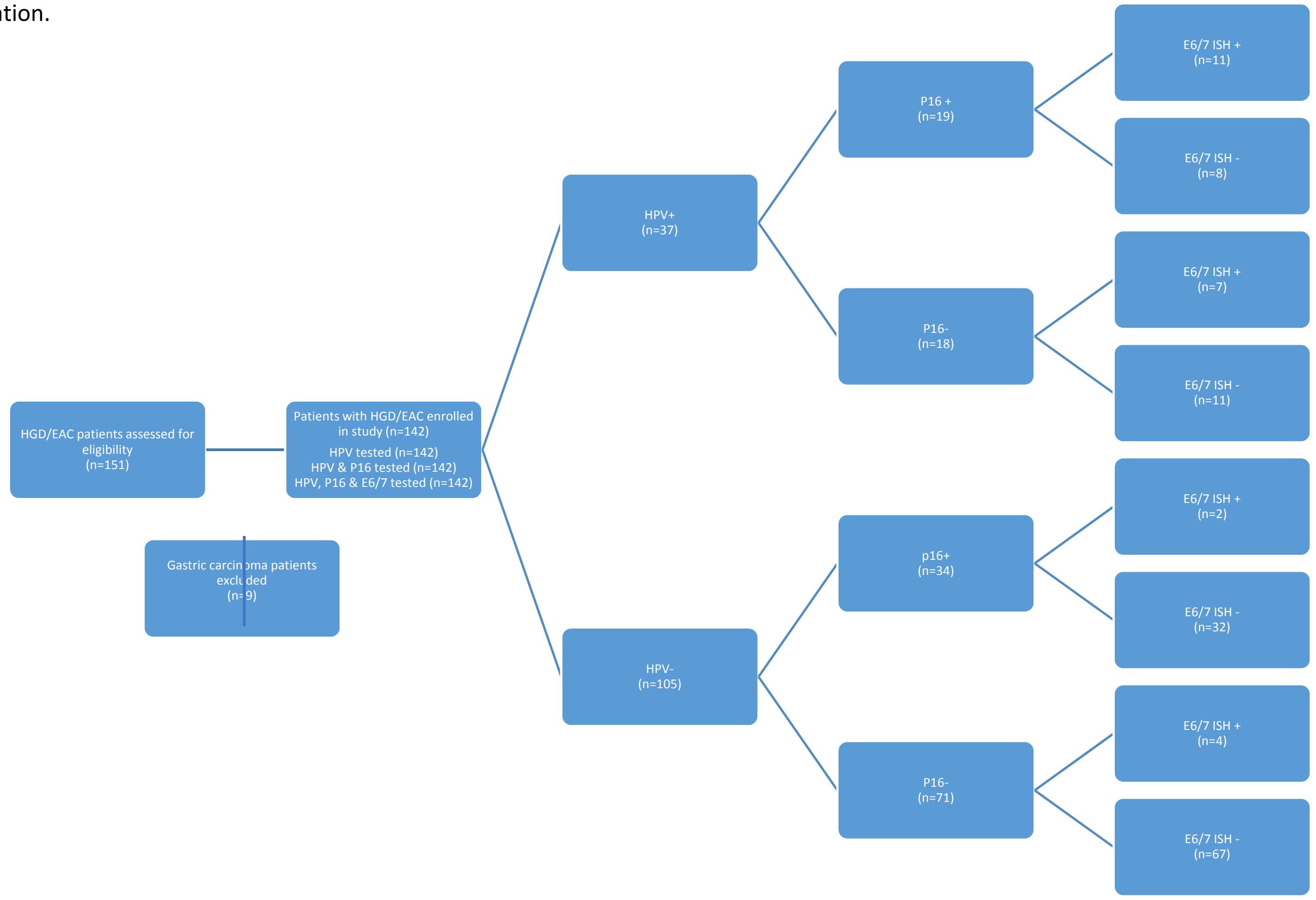

© 2018 Rajendra S et al. *JAMA Network Open.*

eFigure 2. E6 and E7 mRNA Status and p16 Overexpression

Actuarial Kaplan-Meier graphs of survival amongst study patients with high-grade dysplasia/esophageal adenocarcinoma as a function of E6/E7mRNA (A, B) and p16 expression (C, D).

E6/E7 mRNA Status

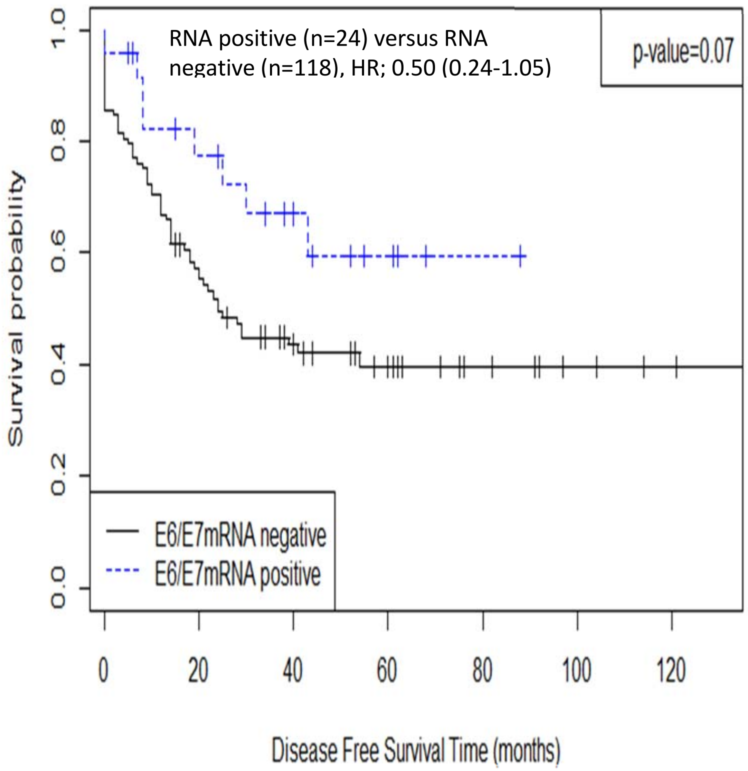

Disease Free Survival Time (Months)

Status	0	20	40	60	80	100	120
Negative	118	65	51	48	48	48	48
Positive	24	19	15	15	15	15	15

eFigure 2A

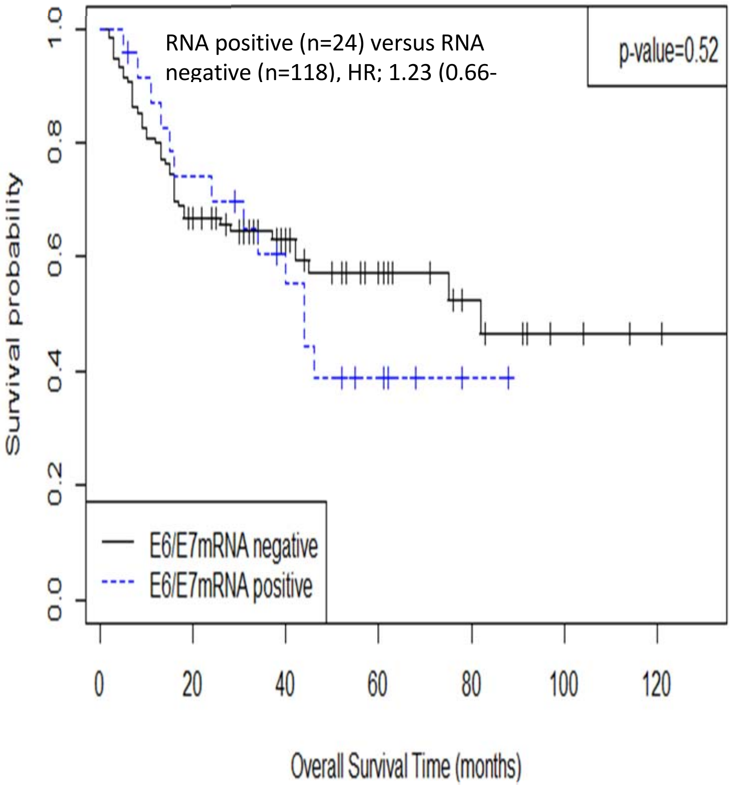

Overall Survival Time (Months)

Status	0	20	40	60	80	100	120
Negative	118	80	75	68	56	56	56
Positive	24	18	14	10	10	10	10

eFigure 2B

© 2018 Rajendra S et al. *JAMA Network Open.*

P16

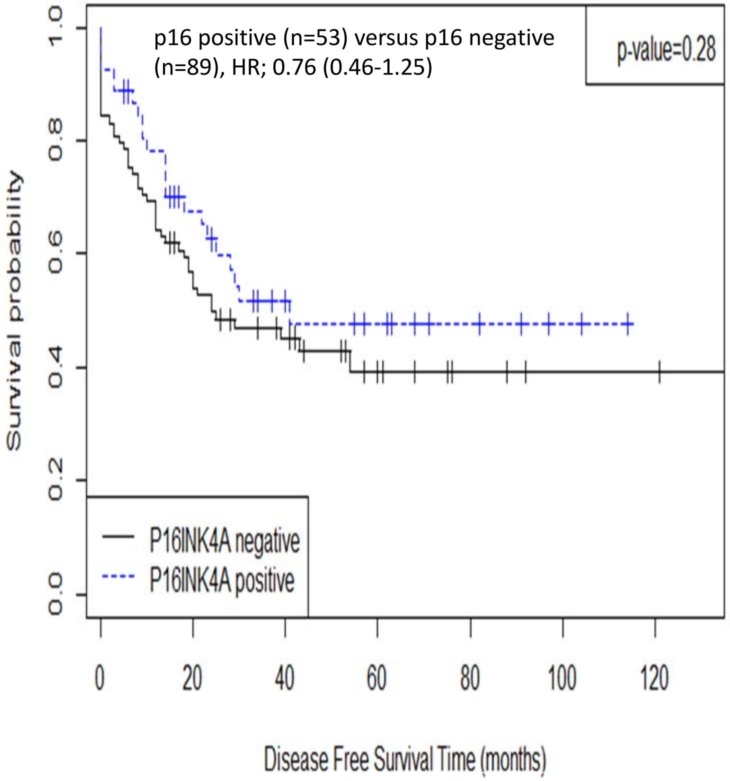

Disease Free Survival Time (Months)

Status	0	20	40	60	80	100	120
Negative	89	49	41	35	35	35	35
Positive	53	37	26	26	26	26	26

eFigure 2C

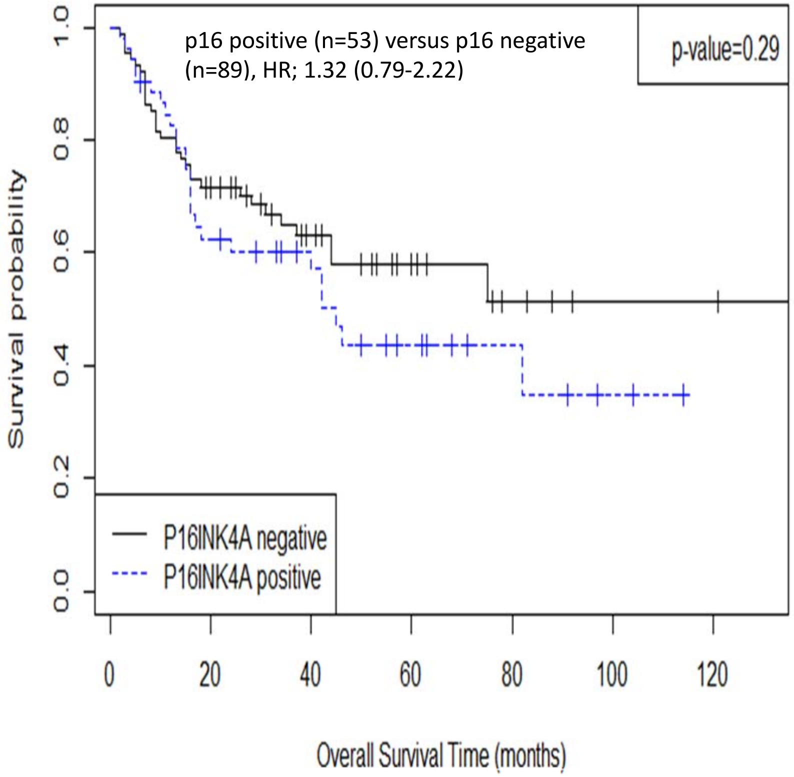

Overall Survival Time (Months)

Status	0	20	40	60	80	100	120
Negative	89	65	57	46	46	46	46
Positive	53	33	31	24	19	19	19

eFigure 2D

© 2018 Rajendra S et al. *JAMA Network Open.*

eFigure 3. P53 Expression

Actuarial Kaplan-Meier graphs of survival amongst study patients with high-grade dysplasia/esophageal adenocarcinoma as a function of p53 expression (A, B) in tumor.

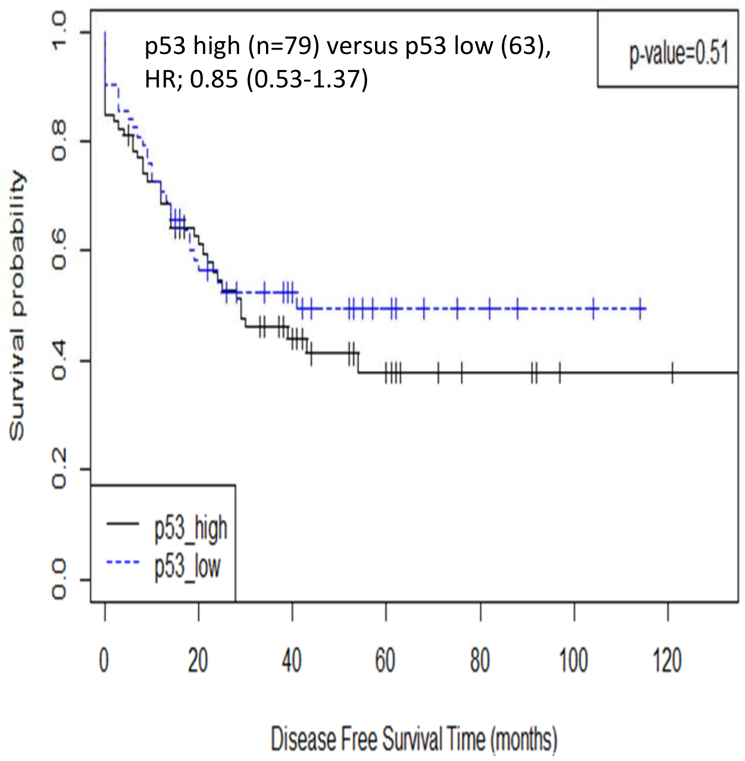

Disease Free Survival Time (Months)

Status	0	20	40	60	80	100	120
High	79	49	35	31	31	31	31
Low	63	36	31	31	31	31	31

eFigure 3A

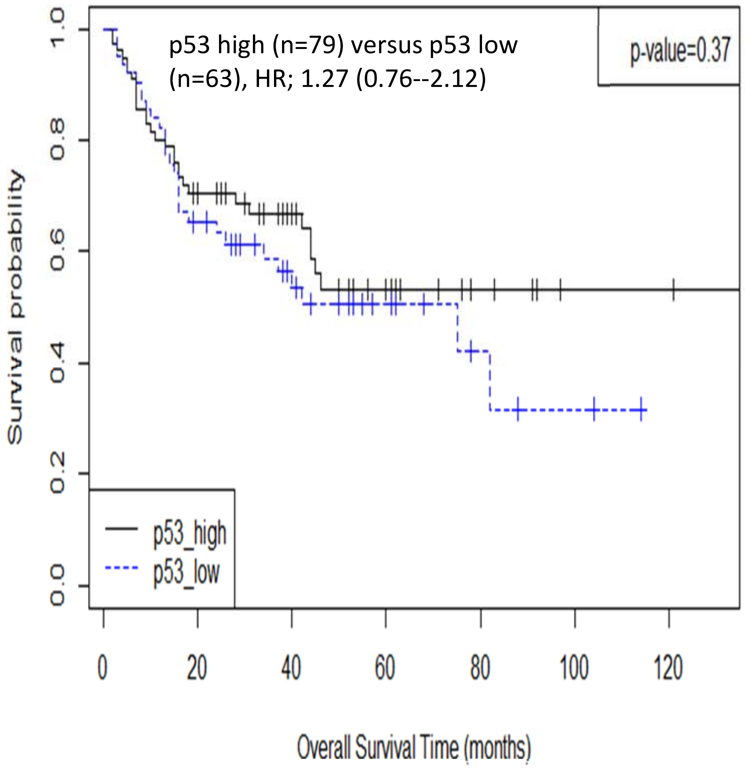

Overall Survival Time (Months)

Status	0	20	40	60	80	100	120
High	79	56	51	42	42	42	42
Low	63	41	35	27	21	21	21

eFigure 3B

© 2018 Rajendra S et al. *JAMA Network Open.*

eTable. Demographic and Clinical Characteristics of the Study Population and Associated Tumors According to Patient Group

Characteristic	**HGD/EAC patients with transcriptionally active HPV** (N=30; 21%)	**HGD/EAC patients without transcriptionally active HPV** (N=112; 79%)	P value[a]
Sex (M/F)	25/30 (83.3%)<br>5/30 (16.7%)	101/112 (90.2%)<br>11/112 (9.8%)	0.29
Age (mean) in years	61.4 (SD=11.9)<br>Range: 33-89	67.2 (SD=11.9)<br>Range: 32-90	0.02
BMI	27.0 (SD+/- 5.7)	27.3 (SD+/-5.1)	0.74
Recurrence or Progression	9/30<br>(30%)	61/112<br>(54%)	0.02
Ever smoked	19 (63%)	82 (73%)	0.29
>10 pack years[b]	17 (89%)	74(91%)	0.80
>20 pack years[b]	11 (58%)	52 (64%)	0.61
Alcohol intake	21 (72.4%)	80 (72.1%)	0.97
Excess Alcohol Intake	6 (20.7%)	20 (18.2%)	0.76
PPI	22 (73.3%)	81 (73.0%)	0.97
NSAID's	7 (23.3%)	27 (24.3%)	0.91
Statins	7 (23.3%)	35 (31.5%)	0.38
Multifocal disease	12/23 (52.2%)	30/81 (37.0%)	0.19
Oesophagitis	2/30 (6.7%)	16/109 (14.7%)	0.25
Hiatus hernia	19/29 (65.5%)	51/107 (47.7%)	0.09
Resection Margin<br>R0<br>R1/R2	 21/27 (77.8%)<br>6/27 (22.2%)	 65/76 (85.5%)<br>11/76 (14.5%)	 0.35
Histology –degree of differentiation<br>(Well differentiated versus not well differentiated)<br><br>Well<br>Moderate<br>Poor<br>Not documented	3 (13.64)<br>11 (50.0%)<br>6 (27.3)<br>2 (9.1%)	5 (6.5%)<br>34 (44.2%)<br>30 (39.0)<br>8 (10.4%)	0.28
Treatment<br>Endotherapy (EMR± RFA)	 13 (43.3%)	 45 (40.5%)	 0.78
Esophagectomy	19 (63.3%)	55 (50.0%)	0.18
Pathologic T stage[c]			

© 2018 Rajendra S et al. *JAMA Network Open.*

Tis/T1/T2<br>T3/T4	20/30 (66.7%)<br>10/30 (33.3%)	65/112 (58.0%)<br>47/112 (42.0%)	0.42
Pathologic N stage[c]<br>N0-N1<br>N2-N3	 26/30 (86.7%)<br>4/30 (13.3%)	 101/112 (91.0%)<br>10/112 (9.0%)	0.48
Pathologic M stage[c]<br>M0<br>M1	 30/30 (100%)<br>0/30 (0%)	 104/112 (93.7%)<br>7/112 (6.3%)	 0.16
Radiotherapy<br>Chemotherapy	7/30 (23.3)<br>10/30 (33.3%)	29/112 (26.1%)<br>41/112 (36.9%)	0.76<br>0.72
Disease free survival (DFS)	39.1 (SD+/- 30.6)	25.6 (SD+/- 27.7)	0.02
Overall survival (OS)	41.6 (SD+/- 29.5)	31.4 (SD+/- 27.4)	0.08
Survival Status (Alive at last follow-up)	15/30 (50%)	69/111 (62.2%)	0.23
Recurrence/Progression	9/30 (30%)	61/112 (54%)	0.02

[a]Differences between HPV positive versus HPV negative cases in regard to baseline characteristics were assessed using two-sample t-test for all numerical data and chi-square analysis for binary measurements.
[b]A pack-year=smoking one packet of cigarettes per day for a year.
[c]TNM Classification as per AJCC Cancer Staging Manual 7th Edition.
Abbreviations: BMI; body mass index, EAC; esophageal adenocarcinoma, EMR; endoscopic mucosal resection, HGD; high-grade dysplasia, HPV; human papillomavirus PPI; proton-pump inhibitors, NSAID's, non-steroidal anti-inflammatory drugs, RFA; radio-frequency ablation

© 2018 Rajendra S et al. *JAMA Network Open.*
